# Osteoarthritis- a systematic review of long-term safety implications for osteoarthritis of the knee

**DOI:** 10.1186/s12891-019-2525-0

**Published:** 2019-04-09

**Authors:** Jonathon Charlesworth, Jane Fitzpatrick, Nirmala Kanthi Panagodage Perera, John Orchard

**Affiliations:** 1 Australasian College of Sport and Exercise Physicians, 257 Collins Street, Melbourne, VIC 3000 Australia; 2 Division of Physiotherapy, Department of Medical and Health Science, Linköping University, Linköping, Sweden; 3 University of Melbourne, Melbourne, Australia; 4 Sports Medicine Professionals, University of Melbourne, Level 7, Alan Gilbert Bdg, 161 Barry Street, Melbourne, VIC 3010 Australia; 5 School of Public Health, University of Sydney, Sydney, NSW Australia

**Keywords:** Osteoarthritis, Knee, Exercise, Injections, Surgery

## Abstract

**Background:**

There is no cure for knee osteoarthritis (KOA) and typically patients live approximately 30-years with the disease. Most common medical treatments result in short-term palliation of symptoms with little consideration of long-term risk. This systematic review aims to appraise the current evidence for the *long-term* (≥12 months) safety of common treatments for knee osteoarthritis (KOA).

**Methods:**

Cochrane Database of Systematic Reviews, Medline and PubMed were systematically searched from 1990 to July 2017, inclusive. Inclusion criteria were 1) peer-reviewed publications investigating treatments for KOA referred to in the Australian Clinical Care Standard and/or Therapeutic Guidelines: Rheumatology 2) specifically addressing safety of the treatments 3) with ≥12 months of follow-up and 4) Downs and Black quality score ≥ 13.

**Results:**

Thirty-four studies fulfilled the inclusion criteria. Lifestyle modifications (moderate exercise and weight loss), paracetamol, glucosamine, Intraarticular Hyaluronic Acid (IAHA) and platelet-rich-plasma (PRP) injections have a low risk of harm and beneficial ≥12 month outcomes. Although Nonsteroidal Anti-inflammatory Drugs (NSAIDs) provide pain relief, they are associated with increased risk of medical complications. Cortisone injections are associated with radiological cartilage degeneration at > 12 months. Arthroscopy for degenerative meniscal tears in KOA leads to a 3-fold increase in total knee arthroplasty (TKA). TKA improves primary outcomes of KOA but has a low rate of significant medical complications.

**Conclusions:**

Given the safety and effectiveness of lifestyle interventions such as weight loss and exercise, these should be advocated in all patients due to the low risk of harm. The use of NSAIDs should be minimized to avoid gastrointestinal complications. Treatment with opioids has a lack of evidence for use and a high risk of long-term harm. The use of IAHA and PRP may provide additional symptomatic benefit without the risk of harm. TKA is associated with significant medical complications but is justified by the efficacy of joint replacement in late-stage disease.

**Trial registration:**

PROSPERO International prospective register for systematic reviews; registration number CRD42017072809.

**Electronic supplementary material:**

The online version of this article (10.1186/s12891-019-2525-0) contains supplementary material, which is available to authorized users.

## Background

Osteoarthritis (OA) is a chronic degenerative joint disease of dynamic pathology with multifactorial aetiology. It involves progressive softening and loss of articular cartilage, subchondral bone sclerosis, cyst formation and the development of osteophytes. OA of the knee accounts for more dependence in walking, stair climbing and other lower-extremity tasks that any other disease [1]. To illustrate the impact of knee OA (KOA) on a typical population in a Western country, we’ve chosen example references from Australia, familiar to our author group. International comparison studies show that Australia’s rate of KOA is similar to other Western nations and affects 2.1 million Australians (9% of the population) [2]. Large numbers of arthroplasties are performed due to KOA; in 2015, 54,277 total knee arthroplasty (TKA) surgeries were performed in Australia due to KOA [3].

The median age of KOA diagnosis is 55-years and typically people live about 30-years with the disease [4]. As there is no curative treatment for OA currently; treatments are aimed at reducing pain and improving function. Systematic reviews (SR) are a useful method to synthesise the efficacy of treatments for KOA, however most of these reviews have not considered the long-term risks associated with treatments. This was because most studies follow patients for a short period of time (e.g. 3–6 months). This results in a significant evidence gap in the literature because it is likely that short-term improvements such as pain relief are overrepresented whilst potential long-term risks might be underrepresented in these studies. Given that a patient with KOA lives with their condition for an average of 30-years, an evidence-based understanding of the safety of treatments is important to ensure patient safety. This review aims to appraise the current evidence for long-term (≥12 months) safety of common treatments for KOA.

## Methods

Three databases (Cochrane Database of Systematic Reviews, Medline and PubMed) were systematically searched from 1990 to July 2017 inclusive, following the Preferred Reporting Items for Systematic Reviews and Meta-analysis (PRISMA) guidelines [5]. A priori study protocol was registered on the PROSPERO International prospective register for systematic reviews (http://www.crd.york.ac.uk/PROSPERO/display_record.php?ID=CRD42017072809); registration number CRD42017072809. The PICO (population, intervention, comparator/control and outcome) concept [6] was used to develop search strategy (Appendix 1) and the inclusion/exclusion criteria (Appendix 2). Studies were considered if they were: 1) peer reviewed publications investigating treatments for KOA listed in the Australian Clinical Care Standard [7] and/or Therapeutic Guidelines: Rheumatology [8], 2) specifically addressed safety of the treatments; 3) with ≥12 months of follow-up and 4) Downs and Black [9] quality score > 13 (Appendix 2). A study was excluded from the review if it failed a single criterion.

The ‘long-term effect’ for KOA treatments was defined as any effect which persisted ≥12 months post-treatment. For treatments that were used on a single occasion and discontinued (e.g. surgery, injections), the follow-up period after treatment was determined to be at least 12 months. With respect to ongoing treatments (e.g. analgesia), an independent measure of disease-modification was required. This was to avoid the issue of repeated short-term effects potentially masking long-term effects of ongoing treatment. Minor side effects and adverse events (e.g. allergic rash, post-procedure soreness) were not included in this review as they are typically short-term issues. However, adverse events such as drug addiction and cardiovascular issues were included as these could persist beyond 12 months. Long-term effect of a treatment could be either:

1. positive OR negative effect of index treatment vs comparison treatment in any trial persisting at ≥12 months; or
2. positive OR negative effect of index treatment vs comparison treatment in any trial showing significant change in any objective disease indicator (e.g. cartilage thickness on magnetic resonance imaging (MRI) or X-ray) at ≥12 months; or
3. effect of index treatment showing significantly increased OR reduced risk of progression to TKA; or
4. significant increase in side-effect or harm related to treatment with a potential for ongoing harm from this condition to persist beyond 12 months

## Results

### Study selection

**Fig. 1:**
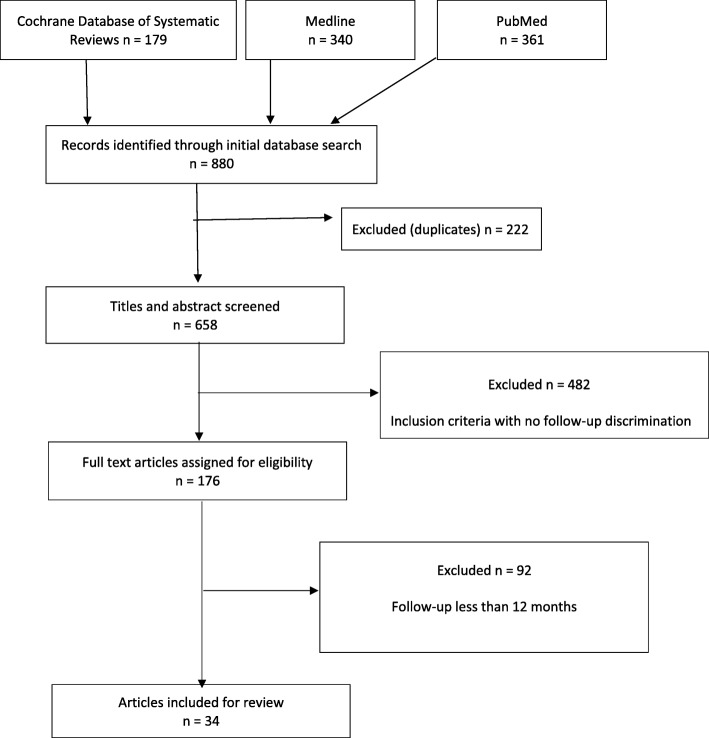
Study selection flow diagram

Of the 880 records identified in the initial keyword search, 34 studies fulfilled the inclusion criteria, had a Downs and Black [9] quality score ≥ 13 (Appendix 2) and are included in the synthesis (Fig. 1). A summary of included studies including study population characteristics, medium-long term treatment impact on the disease and non-disease side effects that have the potential to last 12 months are presented in Table 1.

**Table 1:** Summary of Studies providing overview of the treatment options for knee osteoarthritis

Treatment option	Level of evidence (type of trial)	Reference	Study population characteristic	Intervention	Outcome measure	Medium-long term impact on the disease	Non-disease side effects (with potential to last 12 months)
Lifestyle							
*Exercise*							
	II (RCT)	Messier et al. (2004) [8]	252 patients older than 60 with BMI > 28	Exercise and diet, diet only, exercise only	WOMAC	Positive	1 patient tripped with laceration to forehead
	II (RCT)	Nejati, et al. (2015) [9]	56 patients with grade 2–4 Kellgren-Lawrence (KL)	Stretching and leg exercises	VAS and KOOS	Positive	Nil
	II (RCT)	Jenkinson et al. (2009) [10]	389 patients older than 45 with BMI > 28	Diet and exercise, diet only, exercise only	WOMAC	Positive	Nil
	II (RCT)	Kawasaki et al. (2008) [11]	142 female patients with moderate knee-OA	Exercise with or without glucosamine or risendronate	WOMAC	Neutral	Nil
*Weight reduction*							
	III(Prospective Cohort)	Gersing et al. (2017) [12]	640 patients	Weight loss > 10% or 5–10%	MRI WORMS	Positive	Nil
Pharmacological							
*Glucosamine and Chondroitin sulphate (CS)*							
	II (RCT)	Pavelká et al. (2002) [13]	202 patients aged 45–70	Glucosamine 1500 mg daily for 3 years	WOMAC	Positive	Gastrointestinal side effects
	II (RCT)	Reginster et al. (2001) [14]	212 patients	Glucosamine 1500 mg daily for 3 years	WOMAC	Positive	Gastrointestinal side effects
	II (RCT)	Sawitzke et al. (2008) [15]	572 patients with KL grade 2–3	Glucosamine or chondroitin	JSW	Neutral	Gastrointestinal side effects
*Paracetamol*							
	II (RCT)	Temple et al. (2006) [16]	571 patients with knee or hip OA	Paracetamol or Naproxen for 6 or 12 months	WOMAC	Positive	Hepatic, renal and gastrointestinal side effects
*Non-steroidal anti-inflammatory drugs (NSAIDs)*							
	II (RCT)	Temple et al. (2006) [16]	571 patients with knee or hip OA	Paracetamol or Naproxen for 6 or 12 months	WOMAC	Positive	Hepatic, renal and gastrointestinal side effects
	II (RCT)	Sawitzke et al. (2010) [17]	662 patients with KL grade 2–3	Celecoxib 2oomg for 2 years	WOMAC	Neutral	Gastrointestinal side effects
Injectables							
*Intra-articular (IA) corticosteroids*							
	II (RCT)	McAlindon et al. (2017) [18]	140 patients with KL grade 2–3	Triamcinolone 3 monthly for 2 years	MRI cartilage loss	Negative	Facial flushing, injection site pain
	II (RCT)	Raynauld et al. (2003) [19]	68 patients with KL grade 2–3	Triamcinolone 3 monthly for 2 years	WOMAC	Positive	Nil
*Intra-articular hyaluronic acid (IAHA)*							
	III (Prospective cohort)	Benazzo et al. 2016) [20]	50 patients, older than 40	2 x Hymovis 3 ml a week apart, then again at 6 months	WOMAC, JSW	Positive	Increase in knee pain, HA, flu like symptoms, back pain
	III (Prospective cohort)	Conrozier et al. (2003) [21]	155 patients	3 x intraarticular Hylan GF-20 a week apart	Patient satisfaction and safety	Neutral	Nil
	III (Prospective cohort)	Huskin et al. (2008) [22]	62 patients KL grade 1–2	3 x intraarticular Hylan GF-20 a week apart	WOMAC	Positive	Pain, swelling and synovitis
	II (RCT)	Jorgensen et al. (2010) [23]	337 patients	Intra-articular Hyaluron weekly for 5 weeks	LFI, pain on VAS walking > 50 m	Neutral	
	II (RCT)	Juni et al. (2007) [24]	660 patients	3 intra-articular injections of 3 different HAs	WOMAC	Neutral	Pain, swelling and synovitis
	II (RCT)	Navarro-Sarabia et al. (2011) [25]	306 patients KL grade 2–3	5 intra-articular injections at 6 month intervals	OARSI responder criteria	Positive	Pain, swelling, rash and bleeding
	II (RCT)	Ozturk et al. (2006) [26]	24 patients	3 injections as week apart and then repeat at 6 months with or without triamcinolone	WOMAC	Positive	
	II (RCT)	Vaquerizo et al. (2013) [27]	96 patients	3 x PRGF-Endoret or 1 x Durolane	WOMAC	Positive	
	III (Prospective cohort)	Kearey et al. (2017) [28]	119 patients	Single intra-articular injection of 6-mL hylan G-F	WOMAC	Positive	
	III (Prospective case)	Yan et al. (2015) [29]	110 knees from 95 patients	Single intra-articular injection of 6-mL hylan G-F	Pain VAS and Likert scales	Positive	Self-limiting pain and swelling over injection site
*Platelet Rich Plasma (PRP)*							
	II(RCT)	Filardo et al. (2012) [30]	144 patients	3 injections of single or double spinning PRP	IKDC, EQ-VAS and Tegner scores	Positive	Swelling and pain over injection site
	II (RCT)	Smith (2016) [31]	30 patients	3 weekly leucocyte poor PRP	WOMAC	Positive	Nil
*Stem Cells*							
	III (Prospective case)	Emadedin et al. (2012) [32]	6 patients	Bone marrow MSCs	MRI cartilage mapping	Positive	Nil
	III (Prospective case)	Emadedin et al. (2015) [33]	18 patients	Bone marrow MSCs	WOMAC	Positive	Rash and erythema
	III (Prospective case)	Orozco et al. (2013) [34]	12 patients	Bone marrow MSCs	MRI cartilage mapping	Positive	Nil
	II (RCT)	Vega et al. (2015) [35]	30 patients	Allogenic bone marrow MSCs vs IAHA	MRI cartilage mapping	Positive	Transient joint pain and swelling
	III (Prospective case)	Fodor and Paulseth (2016) [36]	8 knees in 6 patients KL grade 1–3	Adipose derived SVF cells	WOMAC	Positive	Nil
Surgical							
*Arthroscopy*							
	III (Prospective cohort)	Rongen et al. (2017) [37]	4674 patients with 335 meniscectomies	Arthroscopic meniscectomy	Progression to TKA	Negative	Nil
*Total Knee Arthroplasty (TKA)*							
	III (Prospective cohort)	Brophy et al. (2014) [38]	1268 patients	Previous knee surgery	Progression to TKA	Neutral	Nil
	III (Prospective cohort)	El-Galaly et al. (2017) [39]	1421 patients	Post-traumatic fracture TKA	Failure of implant	Neutral	Nil
	II(RCT)	Skou et al. (2015) [40]	95 patients with moderate to severe OA	TKA	KOOS	Positive	Major medical side effects
	III(Matched Cohort)	Ansari et al. (2017) [41]	21 patients with KOA having previous micro fracture	TKA	KSS score	Positive	1 x arthrofibrosis

### Methodological quality

Results of the Downs and Black [9] methodological quality assessment was presented in Additional file 1: Table S1. No study received score ≤ 13. Twenty-two studies [11–21, 25–29, 32, 33, 37, 39, 42] reached the maximum quality score of 22 out of 26 and thus have a low risk of bias. The mean score was 21.

### Overview of the treatment options

#### Lifestyle modification

##### Exercise

Four studies [10–13], including 839 patients investigated exercise as treatment for KOA. Clinically significant improvements in Western Ontario and McMaster Universities Osteoarthritis Index (WOMAC), Visual Analogue Scale (VAS) and Knee injury and Osteoarthritis Outcome Score (KOOS) were found at 1-year with no adverse events (Table 1).

##### Weight reduction

Two studies [12, 14], including 956 patients investigated the role of weight reduction as treatment for KOA and no adverse outcomes were reported. Eighty-two patients who were on a dietary intervention aimed for an average of 5% weight loss over 18 months and subsequently reported an 18% improvement in WOMAC scores [12]. In addition, participants who lost more than 5% of their body weight over 48 months showed significantly lower cartilage degeneration on MRI compared to those in the stable weight group [14] (Table 1).

#### Pharmacological management

##### Glucosamine and chondroitin sulphate

Three studies [13, 15, 17] investigated the efficacy of Glucosamine and Chondroitin Sulphate (CS) in 986 patients with KOA for up to 3 years. Two studies demonstrated clinically significant decreases in WOMAC scores. However, no difference in Joint Space Width (JSW) was evident [17]. Minor gastrointestinal side effects such as dyspepsia were present in both the treatment and placebo groups [13, 15, 17] (Table 1).

##### Paracetamol

One RCT [18] with 571 patients randomised to receive 4 g/day Paracetamol or Naproxen 750 mg/day demonstrated improvement from baseline in WOMAC score at 6 and 12 months. Minor hepatic, renal and gastrointestinal side effects were reported (Table 1).

##### Non-steroidal anti-inflammatory drugs (NSAIDs)

Two studies [18, 19], including 1233 patients investigated the use of non-steroidal anti-inflammatory drugs (NSAIDs) as treatment for KOA. Naproxen treatment was associated with improvement from baseline in WOMAC score at 6 and 12 months however gastrointestinal bleeding was reported [18]. In contrast, 662 patients with Kellgren-Lawrence(KL) grade 2–3 KOA received Celecoxib as treatment and did not report any improvement in WOMAC from baseline [19]. In addition, gastrointestinal and renal side effects were reported in this group [17] (Table 1).

##### Opioids

No studies for opiate treatment met the inclusion criteria for this analysis.

#### Injectable

##### Intra-articular (IA) corticosteroids

Two RCTs [20, 21] with 208 patients used the same treatment protocol of 3 monthly triamcinolone injections for 2-years. The primary outcome measures were WOMAC [21] and cartilage loss on MRI [20]. These studies reported mixed results, while improvement in WOMAC was reported over a 2-year treatment period [21], significantly greater cartilage volume loss and no difference in knee pain was reported [20] (Table 1).

##### Intra-articular hyaluronic acid (IAHA)

Ten studies [22–31], including 1904 patients investigated intra-articular hyaluronic acid (IAHAs) as treatment for KOA using WOMAC scores to evaluate the clinical outcome. Seven studies [22, 24, 27–31] demonstrated clinically significant improvement to WOMAC score from baseline while three studies [23, 25, 26] showed no or insignificant difference in pain and function outcomes. The most common side-effects were self-limiting pain and swelling at the injection site and no major adverse events were reported (Table 1).

##### Platelet Rich plasma (PRP)

Two [32, 33] studies with 174 patients investigated platelet rich plasma (PRP) as treatment for KOA. Both studies reported clinically significant improvement in pain and function with no adverse effects. Pain and swelling were the main side effects (Table 1).

##### Stem cells

Five studies [34–38] with 72 patients investigated stem cells as treatment. Bone marrow mesenchymal stem cells were used in four studies and one study used adipose derived stromal vascular fractions. Improvements in WOMAC [35, 38] or MRI cartilage mapping [34, 36, 37] parameters were reported. Due to small sample size, all of aforementioned studies were under powered, as such results are inconclusive. Side effects included rash, erythema, transient joint pain and swelling (Table 1).

#### Surgical

##### Arthroscopy

One study [39] investigated the progression to TKA in patient who underwent meniscectomy because of degenerative meniscal tears. Of the 335 patients who underwent meniscectomy, 63 progressed to a TKA. This was a threefold increase in risk of progression to a TKA compared to an age matched control group (Table 1).

##### Total knee arthroplasty

Four studies [40–43] investigated the long-term effect of various interventions and the progression to TKA. In one study, ninety-five patients with moderate to severe OA showed significant improvement in KOOS score post TKA [42]. However, compared to the control group, the TKA group had a fourfold increase in complications over the 12-month follow-up period. The other studies showed that previous knee surgery including microfracture leads to an increase in progression to a TKA [43] and post-traumatic KOA requiring TKA has a worse outcome than that for non-traumatic OA [41] (Table 1).

## Discussion

This review is the first to synthesise and evaluate the studies which specifically assessed safety of KOA treatment with a minimum follow-up period of a year. This is important because KOA is diagnosed on average at 55-years of age and typically people live with the disease for about 30-years. Lifestyle modifications (moderate exercise [9–13] and weight loss [12–14]), paracetamol [18], glucosamine [15, 16], IAHA [22–31] and PRP injections [32, 33] found to have a low risk of harm and beneficial treatments outcomes at ≥12 months.

Australian general practitioners (GPs) see 6.2 patents with knee-OA for every 1000 consultations [44]. Within the primary care setting, use of nonpharmacological treatments (e.g. exercise) as a first-line management for KOA is low compared to pharmacological management and the rate of surgical referrals are high [44]. Our results indicate that nonpharmacological treatment such as exercise [10–13] and weight management [12, 14] are effective in management of KOA with minimal adverse effects. Primary care settings provide a great platform to support lifestyle interventions effective in treatment and management of KOA. Therefore, weight loss and exercise should be advocated as part of the treatment in all patients due to the low risk of harm, cost effectiveness as well as associated health benefits. It is important to allocate resources and invest in supporting GPs and other primary health care providers to provide lifestyle interventions to treat and manage KOA at the community level.

Opiates continue to be used to manage pain associated with KOA, irrespective of a large body of evidence questioning the benefits of opioid use [45]. We were unable to evaluate evidence on the effectiveness and safety of long-term (≥ 12 months) opioid therapy for KOA because no studies met the inclusion criteria as the follow-ups of those studies considering safety were < 6 months. This is a concern and a limitation of the available evidence relating to KOA management. A recent systematic review into chronic pain management found that there is insufficient evidence to support the effectiveness of long-term opioid therapy [45]. Opioids provide effective analgesia however, benefits are limited by frequent side effects such as nausea (30%) and vomiting (13%), constipation (23%), dizziness (20%) and somnolence (18%) [46]. Long-term opioid use increases the risk of abuse and addiction [45]. Rates of iatrogenic addiction range from 1 to 26% and have to be carefully delineated from misuse or abuse a more conservative diagnosis of addiction is present in around 8% of those prescribed long-term opiates for chronic pain [47]. In the United States, opioids were prescribed to 15.9% of patients with KOA [48] and there has been a significant increase in opioid prescriptions for persons with KOA [49]. Australia is following a similar pattern [50] and opioid overuse is currently dominating public debate. In the first instance, opioids should be prescribed for a short-term trial basis, as part of a multimodal strategy, with regular review of treatment response and adverse effects. This treatment should perhaps be reserved for those who are unable to have other treatments like TKA because of multiple medical comorbidities.

In terms of other oral analgesics, paracetamol has a more favourable safety profile than NSAIDS but the risk of liver toxicity is not negligible on the recommended dose of up to 4 g/day. Treatment with high dose paracetamol (> 3 g/day) is associated with a greater risk of hospitalisation due to gastrointestinal (GI) perforation, ulceration or bleeding compared to 3 g/day [51]. NSAIDS generally provide superior pain relief compared to paracetamol but with increased safety considerations. For example, long-term use of NSAIDs medications are associated with increased risk of complications such as gastrointestinal [18, 19] and renal [19] complications. Indeed, oral NSAIDs increase in the risk of upper gastrointestinal complications, including peptic ulcer perforation, obstruction, and bleeding by three- to five-fold. The use of selective COX2 inhibitors like celecoxib has been researched extensively to decrease the gastrointestinal risk. However, a recent RCT reported that treatment with celecoxib increased the risk of recurrent GI bleeding by 9% in very high-risk patients [52]. In addition, prolonged NSAID use is associated with adverse cardiovascular events in the short and long term [53–55].

There are risks associated with injectable treatments such as cortisone, IAHA and PRP. Recurrent cortisone injections into the knee decrease cartilage volume [20]. In addition, cortisone injections into the knee before surgery increases the risk of subsequent infection in people who undergo TKA [56]. The incidence of serious infectious complications following cortisone injections into the knee ranges widely, and may be as high as 1 in 3000 in high-risk patients [57]. Systemic effects reported include transient hyperglycemia, warmth or flushing of the skin and cushingoid appearance if treatment is too frequent. Local reactions include subcutaneous or cutaneous atrophy and capsular periarticular calcification [57].

Adverse events associated with IAHAs include severe acute inflammatory reactions (SAIR) or pseudo septic reactions. Whilst 2–8% of patients who received the cross-linked hylan G-F 20 preparation reported a SAIR [58], SAIRs have not been reported after injection of any of the natural IAHAs. Despite similarities, IAHA products should not be treated as a group, as there are differences in IAHA products that influence both efficacy and safety. A recent Cochrane review did not report any major safety issues associated with IAHA except local adverse events such as transient pain and swelling at the injection site [59].

Surgical procedures are associated with risk of infection, bleeding and deep vein thrombosis (DVT). Since there is a tendency for high rates of surgical referrals among Australian GPs [44], it is important to avoid unnecessary surgeries such as knee arthroscopy for degenerative menisci, particularly because that progression to a TKA may be greater after an arthroscopy [39]. In 2014, there were 54, 277 knee replacements performed in Australia [3]. Compared to other Organisation for Economic Co-operation and Development countries, Australia has a higher than average TKA rate. It is generally presumed that the increase in population ageing and obesity rates are responsible for the observed increase in TKA rates. In Australia, 63.4% adults are overweight or obese [60] and 16% Australians are aged 65 years and over [61]. In addition, 45% of the population does not meet physical acitivity reccomendations [60]. Therefore, as discussed above it is important that primary care practitioners use lifestyle modifications such as exercise and weight management as first lines of treatment given their associated benefits in the management of KOA as well as to reduce the burden on the Australian healthcare system associated with inactivity related complications.

Findings from this review indicate that most of the commonly used KOA treatments (based on short-term improvements or traditional treatment protocols) might have harmful effects in the longer-term. It can be hypothesised that some of the recent increase in rates of TKAs observed in Australia might be due to iatrogenic worsening of KOA. The increasing rates of TKA have been previously explained by better availability of the procedure, ageing and increased obesity. However, long-term worsening of KOA outcomes from the overuse of knee arthroscopy, cortisone injections, NSAIDs and opiates, might have contributed to worsening of clinical outcome for KOA patients. Given that on average patents with KOA live 30-years with their condition, long-term safety and disease progression needs to be considered as a vital aspect of treatment regimen than short-term symptom relief. Perspectives on KOA management need to shift from a short-term viewpoint to a long-term one, with improved clinical acumen in prescribing and managing the risks associated with the currently available treatments options.

### Strengths and limitations

High methodological rigour was maintained by developing an a priori study protocol per PRISMA guidelines [5]. This review, therefore, provides a reliable overview of current data pertaining to long term efficacy and safety of KOA treatments. However, by the standards of a systemic review, another author should have screened the articles and this is a limitation to the search strategy.

The number of studies included is limited as only studies addressing the safety of KOA treatment with ≥12 months of follow-up were included. Thus, short term benefits and studies which did not specifically assess safety were excluded. This limitation is acknowledged as there are good studies that look at short and long-term outcomes including side effects of treatment that don’t specifically discuss safety that have been omitted by the search strategy. Furthermore, a complete picture of a treatment should ideally consider both short and long term. However, given patients typically live approximately 30-years with the disease, it is important to develop an evidence-based understanding of the long-term harms of treatments is important to ensure patient safety.

## Conclusion

KOA management algorithms should shift from a short-term viewpoint to a long-term one, with a focus on the long-term safety and efficacy of currently available treatment options, to enable improved clinical acumen in managing KOA.

Many commonly used KOA treatments have harmful effects in the longer-term. NSAIDs increase the risk of gastrointestinal, renal and cardiovascular side effects. We could locate no evidence regarding the risks of long-term harm (addiction, overdose and death) attributable to opiate treatments in patients with KOA. Surgery is associated with risks of medical complications such as deep vein thrombosis and infection however in late-stage disease can be justified by the efficacy of joint replacement; whilst use of knee arthroscopies cannot be justified.

Exercise and weight loss are both safe and effective for long-term treatment and these should be advocated in all patients. To supplement these lifestyle modifications, the judicious use of analgesia, intra-articular injections of cortisone and consideration of hyaluronic acids and platelet-rich plasma are recommended for symptomatic relief in KOA.

## Additional file

Additional file 1: **Tables S1.** Methodological quality ratings of reviewed papers. (DOCX 27 kb)

## Appendix 1

**Table 2:** Narrative review search strategy

Database		Search terms	**Search number**	**Applied filters**
Cochrane Database of Systematic Reviews	Medical condition terms	TX (Osteoarthritis, knee)	1	Human Language filter (English)
	Treatment terms AND safety	Treatments OR Diet therapy[Mesh] OR Drug therapy[Mesh] OR Rehabilitation[Mesh] OR Surgery [Mesh] OR Therapy AND Safety	2	
	Medical condition terms AND treatment terms AND safety	1 AND 2	3	
Medline	Treatment terms	TX (osteoarthritis OR degenerative arthritis OR degenerative joint disease)	1	Human Language filter (English)
	Treatment terms AND safety	Treatment OR Surgery OR Knee replacement OR TKR OR knee arthroscopy OR Tibial Osteotomy OR NSAIDs OR Opiates OR Paracetamol OR Glucosamine OR Cortisone OR Hyaluronan Gel OR PRP OR Stem Cells OR Exercise Program OR Weight Loss AND Safety	2	
	Medical condition terms AND treatment terms AND safety	1 AND 2	3	
PubMed	Medical condition terms	“Osteoarthritis, Knee”[Mesh] OR TITLE-ABS-KEY (osteoarthritis OR degenerative arthritis OR degenerative joint disease)	1	Human Language filter (English)
	Treatment terms AND safety	“Osteoarthritis, Knee/diet therapy”[Mesh] OR “Osteoarthritis, Knee/drug therapy”[Mesh] OR “Osteoarthritis, Knee/prevention and control”[Mesh] OR OR “Osteoarthritis, Knee/rehabilitation”[Mesh] OR “Osteoarthritis, Knee/surgery”[Mesh] OR “Osteoarthritis, Knee/therapy”[Mesh] OR TITLE-ABS-KEY (Treatment OR Surgery OR Knee replacement OR TKR OR knee arthroscopy OR NSAIDs OR Paracetamol OR Glucosamine OR Opiates OR Cortisone OR PRP OR Stem Cells OR Hyaluronan Gel OR Exercise Program) AND “Safety” [MeSH]	2	
	1 AND 2	1 AND 2	3	

Footnote to Supplement 2: TX: All text; *MeSH* Medical Subject Headings

## Appendix 2

**Table 3:** Inclusion/exclusion criteria for the risks and harms associated with commonly used treatment for knee osteoarthritis literature search

	Inclusion criteria	Exclusion criteria	Rationale for this criteria
Publication type	Peer-reviewed original research articles, systematic reviews and meta-analysis only	Non-peer-reviewed articles, newspapers, opinion pieces, editorials, commentaries and letters to the editor. Conference proceedings/abstracts. Book chapters. Downs and Black [7] quality score > 13	The aim of this review was to investigate the risks and harms associated with commonly used treatment for knee osteoarthritis. For reasons of practicality, it was deemed acceptable to include only studies published in peer-reviewed journals.
Language	English language	Non-English	For reasons of practicality, it was deemed acceptable to include only studies published in English.
Population	Knee osteoarthritis in patients 18-years and older	Knee osteoarthritis in patients 17-years and younger	Average age of knee osteoarthritis diagnosis is 55-years and typically people live about 30-years with the disease.
Intervention	Operative and non-operative management of knee osteoarthritis treatments treatments listed in the Australian Clinical Care Standard [5] and/or Therapeutic Guidelines: Rheumatology [6] and specifically addressed safety of the treatments;	Operative and non-operative management of knee osteoarthritis not listed in the Australian Clinical Care Standard [5] and/or Therapeutic Guidelines: Rheumatology [6].	Commonly accepted treatments were sought out and the authors decided upon treatments listed in Australian Clinical Care Standard [5] and/or Therapeutic Guidelines: Rheumatology [6].
Outcome measures	Studies specifically addressed safety of the treatments with ≥12 months of follow-up. Long-term effect of a treatment could be either: (1) Positive OR negative effect of index treatment vs comparison treatment in any trial persisting at ≥12 months; or (2) Positive OR negative effect of index treatment vs comparison treatment in any trial showing significant change in any objective disease indicator (e.g. cartilage thickness on magnetic resonance imaging (MRI) or X-ray) at ≥12 months; or (3) Effect of index treatment showing significantly increased OR reduced risk of progression to TKA; or (4) Significant increase in side-effect or harm related to treatment with a potential for ongoing harm from this condition to persist beyond 12 months.	Short-term effects of osteoarthritis treatments. Side effects and adverse effects were not included in this review as they are typically short-term issues (e.g. allergic rash, post-procedure soreness).	The primary outcomes of interest for this review was to describe the long-term effects of the knee osteoarthritis treatments. As there is no curative treatment for OA currently, treatments are aimed at reducing pain and improving function. The prevalence of osteoarthritis increases with age, particularly after the age of 55-years such that the average person lives 30-years with knee OA. Therefore, it is important to appraise the long-term effects of knee OA treatment.

## Abbreviations

CS: Chondroitin Sulphate
DVT: Deep Vein Thrombosis
GI: Gastrointestinal
GPs: General Practitioners
IA: Intra-articular
IAHA: Intra-articular Hyaluronic Acid
JSW: Joint Space Width
KL: Kellgren Lawrence
KOA: Knee Osteoarthritis PRP Platelet Rich Plasma
KOOS: Knee injury and Osteoarthritis Outcome Score
MRI: Magnetic Resonance Imaging
NSAIDs: Non-steroidal Anti-inflammatories
OA: Osteoarthritis
RCT: Randomised Controlled Trial
TKA: Total Knee Arthroplasty
VAS: Visual Analogue Scale
WOMAC: Western Ontario and McMaster Universities Osteoarthritis Index
